# High-dose inactivated influenza vaccine is associated with cost savings and better outcomes compared to standard-dose inactivated influenza vaccine in Canadian seniors

**DOI:** 10.1080/21645515.2016.1215395

**Published:** 2016-09-26

**Authors:** Debbie L. Becker, Ayman Chit, Carlos A. DiazGranados, Michael Maschio, Eddy Yau, Michael Drummond

**Affiliations:** a Optum, Burlington, Ontario, Canada; b Sanofi Pasteur, Swiftwater, PA, USA; c Leslie Dan School of Pharmacy, University of Toronto, Ontario, Canada; d inVentiv Health Clinical, Burlington, Ontario, Canada; e Centre for Health Economics, University of York, Heslington, York, UK

**Keywords:** cost-utility, cost-effectiveness, high-dose inactivated influenza vaccine, Fluzone, influenza

## Abstract

Seasonal influenza infects approximately 10–20% of Canadians each year, causing an estimated 12,200 hospitalizations and 3,500 deaths annually, mostly occurring in adults ≥65 years old (seniors). A 32,000-participant, randomized controlled clinical trial (FIM12; Clinicaltrials.gov NCT01427309) showed that high-dose inactivated influenza vaccine (IIV-HD) is superior to standard-dose vaccine (SD) in preventing laboratory-confirmed influenza illness in seniors. In this study, we performed a cost-utility analysis (CUA) of IIV-HD versus SD in FIM12 participants from a Canadian perspective. Healthcare resource utilization data collected in FIM12 included: medications, non-routine/urgent care and emergency room visits, and hospitalizations. Unit costs were applied using standard Canadian cost sources to estimate the mean direct medical and societal costs associated with each vaccine (2014 CAD). Clinical illness data from the trial were mapped to quality-of-life data from the literature to estimate differences in effectiveness between vaccines. Time horizon was one influenza season, however, quality-adjusted life-years (QALYs) lost due to death during the study were captured over a lifetime. A probabilistic sensitivity analysis (PSA) was also performed. Average per-participant medical costs were $47 lower and societal costs $60 lower in the IIV-HD arm. Hospitalizations contributed 91% of the total cost and were less frequent in the IIV-HD arm. IIV-HD provided a gain in QALYs and, due to cost savings, dominated SD in the CUA. The PSA indicated that IIV-HD is 89% likely to be cost saving. In Canada, IIV-HD is expected to be a less costly and more effective alternative to SD, driven by a reduction in hospitalizations.

## Introduction

Seasonal influenza infects approximately 10–20% of the Canadian population each year. While most people recover within 7 to 10 days, severe illness can occur which causes an estimated 12,200 hospitalizations and 3,500 deaths annually.^1^ These deaths primarily occur in adults aged 65 and older (herein referred to as seniors) who are more susceptible to downstream complications associated with influenza infection.^2,3^ Because seniors are at greater risk, the National Advisory Committee on Immunization (NACI) encourages all Canadian seniors to receive a vaccination each autumn.^1^

In the Canadian provinces, publicly-funded immunization programs provide free vaccine to all eligible members of the public. Trivalent inactivated influenza vaccine (IIV3) is the current vaccine funded for seniors in most provinces. A new high dose IIV3 vaccine (Fluzone High-Dose; IIV-HD) was approved September 2015 by Health Canada based on evidence from a randomized controlled trial (RCT) demonstrating improvements in efficacy over standard dose IIV3 vaccine (SD) in seniors.^4^ The FIM12 study (Clinicaltrials.gov NCT01427309) showed IIV-HD to be superior to SD in preventing laboratory-confirmed influenza caused by any type or sub-type associated with clinically relevant illness in approximately 32,000 seniors (relative efficacy, 24.2%, 95% CI 9.7–36.5%).^5^ Importantly, the study captured the seasonal variation typical of influenza activity as it was conducted over 2 sequential influenza seasons.^6-9^ The 2011/12 season featured low levels of influenza circulation and a close match between the vaccine and circulating strains,^6^ while the 2012/13 season was more severe with a poor match between vaccine and circulating strains.^7-9^

Although the acquisition cost of the IIV-HD is higher than that of SD, healthcare funding decision-makers need to consider all relevant costs of alternative interventions in relation to the resulting health outcomes. Formal techniques of economic evaluation, such as cost-effectiveness analysis (CEA), can be used to provide valuable information to decision makers regarding efficient allocation of finite healthcare resources. Using detailed data on healthcare utilization (HCU) and clinical outcomes collected from participants in the FIM12 trial, we have previously demonstrated IIV-HD to be a less costly and more effective alternative to SD in a United States (US)-focused cost-utility analysis (CUA) conducted from both a Medicare and societal perspective. Mean per-participant medical costs (in 2014 USD) were lower in the IIV-HD group ($1376.72 USD) than in the SD group ($1492.64; difference –$115.92). Further, the IIV-HD vaccine provided a gain in quality-adjusted life-years (QALYs; mean 8.1502 QALYs/participant) compared with the SD vaccine (8.1499 QALYs/participant) and, due to cost savings driven primarily by fewer hospitalizations among subjects vaccinated with IIV-HD, dominated SD in the CUA.^10^ The objective of the current analysis was to determine if vaccination with the newly approved IIV-HD vs. SD would lead to similar economic benefits in Canada when Canadian unit costs and survival data were considered in place of those used in our US analysis.

## Results

Based on our analysis of HCU, per-participant visits to the emergency room (ER) and non-routine/urgent care visits were slightly higher in the IIV-HD group than in the SD group, and slightly lower for prescription and non-prescription medication use (Table 1, full analysis set). The mean per-participant number of hospitalizations was 0.0937 (1,498 hospitalizations in 15,990 participants) in the IIV-HD group and 0.1017 (1,629 hospitalizations in 15,993 participants) in the SD group, with averages of 0.4869 d and 0.5626 d for hospital length of stay (LOS) per-participant, respectively.

**Table 1.** Outcomes per vaccine.

	Full analysis set			Cardiorespiratory condition analysis set		
Outcomes	IIV-HD vaccine n = 15,990	SD vaccine n = 15,993	Difference (IIV-HD-SD)	IIV-HD vaccine n = 15,990	SD vaccine n = 15,993	Difference (IIV-HD-SD)
Uses of prescription medications	0.1977	0.1985	−0.0008	0.0085	0.0087	−0.0002
Uses of non-prescription medications	0.1811	0.1839	−0.0028	0.0061	0.0081	−0.0020
ER visits	0.0131	0.0128	0.0003	0.0004	0.0003	0.0001
Non-routine/urgent care visits	0.2257	0.2179	0.0078	0.0071	0.0074	−0.0003
Hospitalizations	0.0937	0.1017	−0.0080	0.0248	0.0296	−0.0048
Deaths	0.0052	0.0053	−0.0001	0.0016	0.0025	−0.0009
Life-years	9.8505	9.8502	0.0003	9.8851	9.8762	0.0089
QALYs	7.5533	7.5530	0.0003	7.5882	7.5813	0.0069

Ninety-one percent of the total healthcare payer costs and 76% of the total societal costs were due to hospital admissions. The total costs per participant were $60 higher in the SD group compared to the IIV-HD group. Further, utilizing IIV-HD instead of SD, representing an additional expenditure of $25.97/participant, yielded a 181% financial return ($47.15/participant) to the healthcare system mainly through reductions in hospital admissions (Table 2).

**Table 2.** Costs by resource item (in Canadian dollars/participant).

	Full analysis set			Cardiorespiratory condition analysis set		
Outcomes	IIV-HD vaccine n = 15,990	SD vaccine n = 15,993	Difference (IIV-HD-SD)	IIV-HD vaccine n = 15,990	SD vaccine n = 15,993	Difference (IIV-HD-SD)
Study vaccine	$31.81	$5.84	$25.97	$31.81	$5.84	$25.97
Prescription medications	$3.45	$3.45	$0.00	$0.18	$0.19	−$0.01
ER visits	$6.63	$6.47	$0.16	$0.19	$0.13	$0.06
Non-routine/ urgent care visits	$19.21	$18.55	$0.66	$0.60	$0.63	−$0.03
Hospitalizations	$616.47	$690.42	−$73.95	$170.61	$217.26	−$46.65
Total healthcare payer costs	$677.57	$724.72	−$47.15	$203.40	$224.05	−$20.65
Non-prescription medications	$0.32	$0.34	−$0.02	$0.01	$0.01	$0.00
Productivity losses	$136.72	$149.45	−$12.73	$27.28	$35.94	−$8.66
Total societal costs	$814.61	$874.52	−$59.91	$230.70	$259.99	−$29.29

The QALY analysis predicted that IIV-HD recipients would experience 7.5533 QALYs over the remainder of their lifetime, whereas SD recipients would have 7.5530 QALYs, a difference of 0.0003 QALYs in favor of IIV-HD (Table 1 and Table 3). For a cohort the size of the IIV-HD recipients in the clinical trial (N = 15,990), this equates to an additional 4.8 QALYs for the cohort. Since total costs were lower in the IIV-HD group and the health outcomes were more favorable for IIV-HD, the cost-effectiveness analysis found that IIV-HD dominated (i.e., IIV-HD provided more health at lower costs) SD vaccine from both the public payer and societal perspectives. A threshold analysis determined that vaccination with IIV-HD continued to be cost-saving to the public payer up to a IIV-HD price of approximately $79 per injection. IIV-HD remained dominant in the 2 sub-group analyses in study participants with one or more comorbid conditions and in participants ≥75 years (societal perspective only). In the public payer perspective analysis involving the subgroup of participants ≥75 years, a small incremental cost-effectiveness ratio (ICER) of $82/QALY gained was obtained due to slightly higher costs (<$1) and QALYs (0.0049) among participants in the IIV HD group compared to the SD group (Table 3).

**Table 3.** Cost utility analysis.

		Full analysis set					Cardiorespiratory condition analysis set				
Population	Treatment group	Cost /Subject ($CDN)	Difference in cost ($CDN)	QALYs /Subject	Difference in QALYs	ICER (cost/QALY)	Cost /Subject ($CDN)	Difference in cost ($CDN)	QALYs /Subject	Difference in QALYs	ICER (cost/QALY)
Public Payer perspective											
All subjects	IIV-HD (n = 15,990)	$678		7.5533			$203		7.5882		
	SD (n = 15,993)	$725	$47	7.5530	−0.0003	Dominated	$224	$21	7.5813	−0.0069	Dominated
Subjects with 1+ comorbid conditions	IIV-HD (n = 10,750)	$831		7.5411			$257		7.5853		
	SD (n = 10,752)	$876	$45	7.5399	−0.0012	Dominated	$293	$36	7.5750	−0.0103	Dominated
Subjects 75 + years of age	IIV-HD (n = 5,409)	$892		7.5242			$265		7.5807		
	SD (n = 5,430)	$892	<$1	7.5194	0.0049	$82	$292	$27	7.5659	−0.0148	Dominated
Societal perspective											
All subjects	IIV-HD (n = 15,990)	$815		7.5533			$231		7.5882		
	SD (n = 15,993)	$875	$60	7.5530	−0.0003	Dominated	$260	$29	7.5813	−0.0069	Dominated
Subjects with 1+ comorbid conditions	IIV-HD (n = 10,750)	$996		7.5411			$292		7.5853		
	SD (n = 10,752)	$1,055	$59	7.5399	−0.0012	Dominated	$340	$47	7.5750	−0.0103	Dominated
Subjects 75 + years of age	IIV-HD (n = 5,409)	$1,062		7.5242			$303		7.5807		
	SD (n = 5,430)	$1,072	$10	7.5194	−0.0049	Dominated	$340	$37	7.5659	−0.0148	Dominated

**Table 4.** Unit Costs of Resource Items (2014 Canadian dollars).

Resource Item	Cost/Unit	Reference
IIV-HD (Fluzone® High-Dose) vaccine	$31.823/injection	[15]
SD vaccine	$5.82/injection	Sanofi Pasteur
Ibuprofen, 200 mg, 3 and a half times daily for 4 d (non-prescription)	$1.54/course	[19]
Ibuprofen, 600 mg, 3 times daily for 4 d (prescription)	$10.53/prescription	[17]
Oseltamivir, 75 mg, twice daily for 5 d (prescription)	$52.45/prescription	[17]
Azithromycin, 500 mg day 1, 250 mg days 2–5 (prescription)	$17.30/prescription	[17]
Emergency department visit	$506.53/visit	[20, 21]
Non-routine medical office / urgent care visit	$85.13/visit	[21]
Hospitalization per diems	dependent on ICD-10 code (n = 146)	[22]
Daily wage	$188.40/day	[24]

In the cardiorespiratory condition analysis IIV-HD recipients gained more QALYs over their lifetime than SD recipients and their healthcare system and societal costs were also less (Tables 1-3). The total costs per participant were $29 lower in the IIV-HD group than they were in the SD group. The expenditure required to administer IIV-HD instead of SD ($25.97/participant) yielded an 80% financial return ($20.65/participant) to the healthcare system (Table 2), and vaccination with IIV-SD remained cost-saving up to a cost per injection of approximately $53. Further, IIV-HD remained dominant in the cost-effectiveness analysis including all sub-group analysis (Table 3).

**Figure 1.**
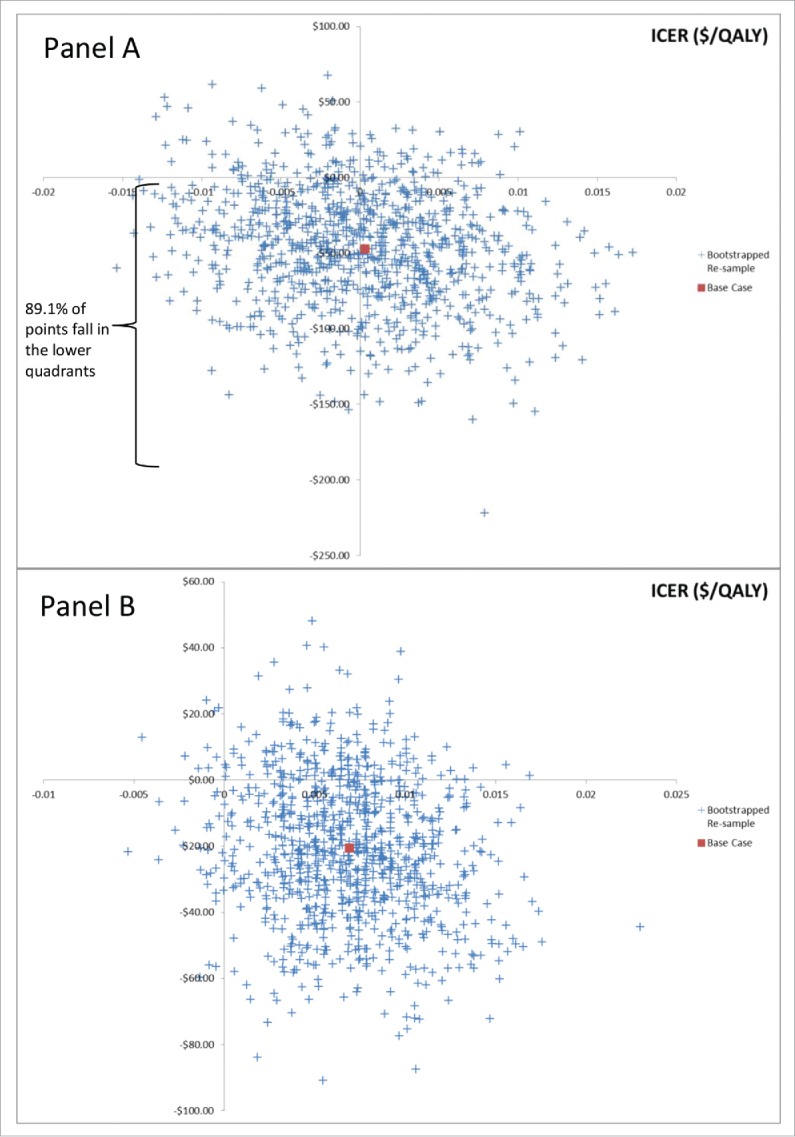
Scatter plots representing the statistical uncertainty through 1,000 bootstrapped samples. Panel A) represents the full analysis set. 89% of bootstrapped data showed that IIV-HD was cost-saving. Panel B) represents data from the cardio-respiratory condition analysis set. 80% of the bootstrapped data showed that IIV-HD was cost-saving and more effective.

ICERs computed in the PSA revealed that 89% of the points in the full-analysis set and 83% of the points in the cardiorespiratory analysis set clustered in the lower quadrants of the plot (Fig. 1). Further, in the cardiorespiratory set 80% of the samples clustered in the lower right quadrant. These results illustrate that IIV-HD is less costly than SD with high certainty. The cardiorespiratory analysis also illustrates that reduction in cardio-respiratory complications provide much of the health benefits offered by IIV-HD.

## Discussion

In this Canadian economic evaluation based on a RCT of approximately 32,000 seniors, we found that IIV-HD was a cost-saving alternative to SD, the current standard of care for ambulatory seniors in most provinces, and this conclusion was robust in the face of statistical uncertainty. From the societal perspective, savings were estimated to be $60 per IIV-HD participant when considering the full analysis set and $29 per IIV-HD participant when considering the cardiorespiratory condition analysis set. Savings to the healthcare payer were slightly less, at approximately $47 and $21 per IIV-HD participant, respectively. Since the price of IIV-HD has not been established in Canada yet, we used the US price assuming a 1:1 exchange rate (based on exchange rates current at the time the study was conducted; $31.81 per injection); however, the actual Canadian launch price will depend on a number of factors such as, for example, global supply and demand constraints and changes in exchange rates. Therefore, we conducted threshold analyses on IIV-HD price which determined that the price could increase to just under $79 (full analysis set) and $53 (cardiorespiratory condition analysis set) per injection and still be cost saving to healthcare payers. As noted, the majority of the savings generated by IIV-HD were driven by reductions in cardiorespiratory hospitalizations plausibly related to influenza.

Our previous analysis based on the same RCT, but conducted from the Medicare and societal perspectives in the US, found similar results, though the magnitude of the cost savings was higher in the US (e.g., $128 and $80 USD (2014) per IIV-HD participant based on the full and cardiorespiratory condition analysis sets, respectively). This difference was driven by the fact that hospital per diems were, on average, approximately 51% (or ∼$1,400/day without adjustment for exchange rate) lower in Canada than in the US.^10^ The only other IIV-HD cost-effectiveness study identified in the literature was a mathematical modeling study (discussed in depth in our previous publication^10^) which predicted that the IIV-HD would be a cost-effective alternative to SD (incremental cost-effectiveness ratio $5,399 USD/QALY gained).^11^

A key feature of this study is that it uses a randomized head-to-head design (FIM12), to define causality between the vaccines and healthcare resource consumption. The FIM12 RCT was sufficiently large to observe the cost-saving signal generated by IIV-HD from background noise (heterogeneous healthcare expenditures among seniors). Further, the healthcare cost data we used are directly applicable to Canadian public payers as they were obtained from Ontario sources, which is the largest province by population in Canada.

The study also had a number of limitations beyond those previously discussed.^10^ As noted, FIM12 was a multinational study, however, only 5% of participants were enrolled in Canada while the remaining 95% of participants were from the US. For our analysis, all HCU data were pooled across all patients even though it is conceivable that HCU may potentially have been affected by differences in the structure of healthcare delivery systems, practice patterns, and the availability and access to healthcare services between the 2 countries. Statistical testing was conducted prior to data pooling, which did not detect heterogeneity among HCU between countries. Therefore, it was justified to pool the trial data for all analyses.

Another limitation is that the collection of per diems for assignment to each hospitalization required some simplifying techniques to manage the data collection burden. As has been explained, hospitalizations were categorized into a manageable number of groups using the Medical Dictionary for Regulatory Activities (MedDRA) coding system, which were first mapped to an International Classification of Diseases, Ninth Edition, Clinical Revision (ICD-9-CM) code(s) and then, specifically for the Canadian analysis, to an ICD Tenth Edition (ICD-10) code(s) for per diem cost collection and assignment from the Ontario Case Costing Initiative (OCCI) database. Although this approach was used consistently across all hospitalizations regardless of vaccine group, it is possible that some specificity was lost during the grouping and 2-step mapping process. To limit the potential for error and/or inaccurate assignment of codes, the same analyst with medical training completed both steps of the mapping exercise, and all mappings were reviewed by a physician at Sanofi Pasteur.

Finally, it is worth noting that our study was a comparison of vaccination with IIV-HD against SD, which is the current standard of care for seniors in most Canadian provinces. However, other vaccines such as standard dose quadrivalent inactivated influenza vaccine and adjuvanted trivalent inactivated influenza vaccine are used for seniors in some provinces and long-term care facilities. Since our analysis was based on data collected in a head-to-head trial comparing only IIV-HD versus SD, we did not consider the cost-effectiveness of IIV-HD compared to other vaccines.

In conclusion, after accounting for the price difference between IIV-HD and SD, vaccination with IIV-HD resulted in cost savings to the public payer and to society in Canada. This was driven by a reduction in the number of hospitalizations. As the clinical benefits are higher for IIV-HD and associated total costs are lower, it dominated SD in the CUA.

## Patients and methods

### Analytic approach

The methods developed for this analysis of the economic impact of IIV-HD vs. SD in Canada as measured in the FIM12 trial^5^ have been described in detail in a previous publication of a CUA conducted for the US.^10^ Here we provide a general overview of these analytic methods along with a detailed description of the adaptations made to the analysis specifically for the Canadian setting.

Briefly, FIM12 was a head-to-head RCT of IIV-HD versus SD (randomized 1:1) that enrolled approximately 32,000 seniors over 2 influenza seasons (2011/2012 and 2012/2013). A surveillance program captured HCU data for all study participants, including use of prescription and non-prescription medications (limited to antipyretics/analgesics/non-steroidal anti-inflammatory drugs, antivirals, and antibiotics), emergency room ER visits, non-routine/urgent care visits, and hospitalizations, if they occurred within 30 d after any study respiratory illness (the frequency and types of respiratory illnesses have been described in the original trial publication^5^). In addition, hospitalizations resulting from serious events (serious events were defined as events: leading to death or hospitalization (or its prolongation); considered as life-threatening or medically important; or resulting in disability) were captured for all participants and for the duration of the study, regardless of their occurrence in relation to a study respiratory illness.^5^

Our primary analysis estimated Canadian public healthcare system expenditures based directly on the HCU data collected in each arm of the FIM12 study. In exploratory analyses, we also examined differences in costs not covered by the public payer, including out-of-pocket costs for non-prescription medications and work force productivity losses. Further, we estimated the incremental cost-utility of IIV-HD vs. SD. To perform the CUA, we modeled the expected impacts of the medical events captured in FIM12 on quality of life (QoL) during the clinical trial period and throughout the life expectancy of the study cohort. Modeled health outcomes that extended beyond the study duration were discounted at 5 percent annually; costs were not discounted since they were based only on HCU reported during the trial period and did not extend beyond one year.^12^ All costs were reported in 2014 Canadian dollars.

### Costing analysis

Although FIM12 was a multinational study, only 5% of participants were enrolled in Canada while the remaining 95% of participants were from the US. Prior to pooling HCU data across all participants, tests of significance were performed to detect potential differences in utilization patterns between the US versus Canada. Specifically, we assessed the impact of vaccine strategy on the total intensity of HCU across countries using the total medical cost, in a single currency (2014 USD), for each subject as a proxy measure for overall HCU intensity. A regression analysis, using a gamma regression model, was conducted with HCU intensity (cost) as the dependent measure and the independent measures were vaccine group, country, and an interaction term between vaccine group and country. It is the interaction term, or whether there is a similar effect of study treatment on the incremental cost difference across countries, that determines whether it is appropriate to pool all participants in a straightforward way.^13^ Because there was not a significant interaction between vaccine group and country with outcome considered to be the total medical cost (p = 0.96; i.e., heterogeneity among HCU was not detected between countries), it was justified to pool the trial data for all analyses.

The pooled healthcare resources for all subjects in the trial were valued using unit prices relevant to Canada from standard sources. In cases where costs were not available in 2014 dollars, they were inflated using the healthcare component of the consumer price index (CPI).^14^ Total costs were calculated from the quantity of resources consumed (for example, number of days hospitalized) and the unit cost per resource. The analysis from the public payer perspective considered the following costs: vaccine, prescription medication, medical visits, and hospitalizations. The analysis from the societal perspective additionally included the costs of non-prescription medication and lost work force productivity.

Since IIV-HD was not marketed in Canada at the time of the study, it was assumed the list price would be the same as in the US (since the USD and Canadian dollar were approximately at par when the study was conducted (2013/2014)) obtained from the Centers for Medicare & Medicaid Services (CMS) List.^15^ The cost of SD was provided by Sanofi Pasteur. The cost per unit for each prescription medication was obtained from the Ontario Drug Benefit Formulary (used as a proxy for Canada).^16^ The cost per prescription included an 8% upcharge and $8.83 dispensing fee per length of treatment.^17^ The unit cost for non-prescription medications (societal perspective only) was obtained from a large Canadian pharmacy chain (visit to Shoppers Drug Mart, Burlington, Ontario, October 2014).

The unit costs for respiratory illness-related ER and non-routine/urgent care visits were obtained from Canadian Institute for Health Information (CIHI) reports.^18,19^ The costs of hospitalizations were calculated by multiplying the hospital LOS by the unit cost per day (per diem). To estimate the per diem cost, diagnosis codes related to hospitalizations were grouped into preferred terms under the MedDRA coding system and mapped to the ICD-9-CM codes (for the purpose of assigning costs in the original US analysis). The ICD-9-CM codes were then mapped to ICD-10 codes for the purpose of extracting and assigning per diem costs from the OCCI's on-line database.^20^

Productivity costs were imputed using methods described by Molinari et al. in which the number of medical visits per participant represented the number of days of productivity lost (at a ratio of one visit: one day of lost productivity).^21^ The number of days of lost productivity was valued at the average daily wage for Canadian employees in 2014.^22^ All unit costs used in the analysis are provided in Table 4.

### Cost-effectiveness

An ICER was calculated for IIV-HD vs. SD. The ICER was defined as (Cost_IIV-HD_ - Cost_SD_)/(Outcomes_IIV-HD_ - Outcomes_SD_). The costs and outcomes were the total costs and outcomes from each arm of the FIM12 trial. We also calculated ICERs for the following 2 subgroups: participants with one or more co-morbid condition (N = 21,502) and participants ≥75 years of age (N = 10,839).

The outcome of the CUA was the QALY. To estimate the number of QALYs in each vaccine arm we first estimated the total number of life-years (LYs) per arm. We used age- and gender-specific Canadian life expectancy data from Statistics Canada to estimate the LYs for study participants.^23^ When weighted according to the FIM12 gender distribution (43% male, 57% female), the mean life expectancy for subjects in FIM12, prior to adjustments for discounting and deaths experienced during the trial was 9.9 y. After applying an annual discount rate of 5%, mean life expectancy for subjects in FIM12 was 7.6 y. For each subject who died during the study, their remaining length of life was calculated based only on the amount of time for which they survived following vaccination (date of death - date of vaccination + 1).

Since QoL data were not collected in the FIM12 trial, we adjusted the LYs to estimate QALYs using QoL data specific to the participant's age, gender, and the medical events they experienced during the study. Additional details regarding the methodology including the utility and disutility values applied in our analysis have been reported in our previously published CUA.^10^

### Subgroup and uncertainty analysis

As FIM12 was not powered for the purpose of this economic evaluation, it was possible that the economic benefit of IIV-HD would be difficult to detect due to the multiple non-influenza related events collected in the FIM12 study. To overcome this and to increase the specificity of our analysis we planned a cardiorespiratory condition analysis that accounted for a subset of clinical outcomes selected by study clinicians before unblinding the study based on the plausibility of their relation to influenza. Outcomes groupings included pneumonia events, asthma/chronic obstructive pulmonary disease/bronchial events, influenza events (serious laboratory-confirmed influenza diagnosed outside study procedures by a participant's healthcare provider), other respiratory events, coronary artery events, congestive heart failure events, and cerebrovascular events. A more granular listing of the conditions included in this subgroup and the methods used to identify these events has previously been published by DiazGranados et al. 2015.^24^

To explore the impact of statistical uncertainty on the results we conducted a bootstrapping analysis of the trial data with replacement as described in our previous work.^10^ Results are presented as a scatterplot on a cost-effectiveness plane.^25^
